# 叶酸调控DUSP1甲基化对非小细胞肺癌细胞奥西替尼耐药性的影响

**DOI:** 10.3779/j.issn.1009-3419.2023.106.24

**Published:** 2023-12-20

**Authors:** Wenjuan HE, Li LIU

**Affiliations:** 430030 武汉，武汉市第四医院药学部; Department of Pharmacy, Wuhan No. 4 Hospital, Wuhan 430030, China

**Keywords:** 叶酸, 双特异性磷酸酶1, 甲基化, 奥西替尼, 肺肿瘤, 耐药性, Folic acid, Dual specificity phosphatase 1, Methylation, Osimertinib, Lung neoplasms, Resistance

## Abstract

**背景与目的** 耐药的产生是肺癌死亡率居高不下的主要原因，本研究旨在探究叶酸（folic acid, FA）调控双特异性磷酸酶1（dual specificity phosphatase 1, DUSP1）甲基化对非小细胞肺癌（non-small cell lung cancer, NSCLC）细胞奥西替尼（Osimertinib, OSM）耐药性的影响。**方法** 浓度梯度递增法建立OSM耐药NSCLC细胞株PC9R。将PC9R细胞随机分为对照组、OSM组（5 μmol/L OSM）、FA组（600 nmol/L FA）、甲基化抑制剂地西他滨组（Decitabine, DAC）（10 μmol/L DAC）、FA+OSM组（600 nmol/L FA+5 μmol/L OSM）和FA+OSM+DAC组（600 nmol/L FA+5 μmol/L OSM+10 μmol/L DAC）。CCK-8法检测细胞增殖能力。划痕实验检测细胞迁移能力。Transwell实验检测细胞侵袭能力。流式细胞术测定分析各组细胞凋亡率。实时荧光定量聚合酶链式反应（real-time fluorescence quantitative polymerase chain reaction, RT-qPCR）法检测细胞DUSP1 mRNA的表达水平。甲基化特异性PCR（methylation specific PCR, MSP）检测各组细胞中DUSP1启动子区甲基化状态。Western blot分析各组细胞DUSP1蛋白、DUSP1下游丝裂原活化蛋白激酶（mitogen-activated protein kinase, MAPK）信号通路关键蛋白的表达水平。**结果** 与对照组相比，OSM组细胞OD_450_值（48、72 h）、划痕愈合率、细胞侵袭数目、DUSP1表达显著下降（P<0.05），细胞凋亡率、DUSP1甲基化水平、p38 MAPK蛋白表达、细胞外调节蛋白激酶（extracellular regulated protein kinases, ERK）磷酸化水平显著上升（P<0.05）；DAC组细胞OD_450_值（48、72 h）、划痕愈合率、细胞侵袭数目、DUSP1表达显著上升（P<0.05），细胞凋亡率、p38 MAPK蛋白表达、ERK磷酸化水平、DUSP1甲基化水平显著下降（P<0.05）。与OSM组相比，FA+OSM组细胞OD_450_值（48、72 h）、划痕愈合率、细胞侵袭数目、DUSP1表达显著下降（P<0.05），细胞凋亡率、DUSP1甲基化水平、p38 MAPK蛋白表达、ERK磷酸化水平显著上升（P<0.05）。与FA+OSM组相比，FA+OSM+DAC组细胞OD_450_值（48、72 h）、划痕愈合率、细胞侵袭数目、DUSP1水平显著升高，细胞凋亡率、DUSP1甲基化水平、p38 MAPK蛋白表达、ERK磷酸化水平显著降低（P<0.05）。**结论** FA可能通过增强DUSP1甲基化抑制DUSP1表达，调控下游MAPK信号通路，进而促进细胞凋亡，抑制细胞侵袭转移，最终减弱NSCLC细胞OSM耐药性。

## Abstract (en)

**Background and objective** Drug resistance is the main cause of high mortality of lung cancer. This study was conducted to investigate the effect of folic acid (FA) on the resistance of non-small cell lung cancer (NSCLC) cells to Osimertinib (OSM) by regulating the methylation of dual specificity phosphatase 1 (DUSP1). **Methods** The OSM resistant NSCLC cell line PC9R was establishd by gradually escalation of OSM concentration in PC9 cells. PC9R cells were randomly grouped into Control group, OSM group (5 μmol/L OSM), FA group (600 nmol/L FA), methylation inhibitor decitabine (DAC) group (10 μmol/L DAC), FA+OSM group (600 nmol/L FA+5 μmol/L OSM), and FA+OSM+DAC group (600 nmol/L FA+5 μmol/L OSM+10 μmol/L DAC). CCK-8 method was applied to detect cell proliferation ability. Scratch test was applied to test the ability of cell migration. Transwell assay was applied to detect cell invasion ability. Flow cytometry was applied to measure and analyze the apoptosis rate of cells in each group. Real-time fluorescence quantitative polymerase chain reaction (RT-qPCR) method was applied to detect the expression level of DUSP1 mRNA in cells. Methylation specific PCR (MSP) was applied to detect the methylation status of the DUSP1 promoter region in each group. Western blot was applied to analyze the expression levels of DUSP1 protein and key proteins in the DUSP1 downstream mitogen-activated protein kinase (MAPK) signaling pathway in each group. **Results** Compared with the Control group, the cell OD_450_ values (48 h, 72 h), scratch healing rate, number of cell invasions, and expression of DUSP1 in the OSM group were obviously decreased (P<0.05); the apoptosis rate, the methylation level of DUSP1, the expression of p38 MAPK protein, and the phosphorylation level of extracellular regulated protein kinases (ERK) were obviously increased (P<0.05); the cell OD_450_ values (48, 72 h), scratch healing rate, number of cell invasions, and expression of DUSP1 in the DAC group were obviously increased (P<0.05); the apoptosis rate, the expression of p38 MAPK protein, the phosphorylation level of ERK, and the methylation level of DUSP1 were obviously reduced (P<0.05). Compared with the OSM group, the cell OD_450 _values (48, 72 h), scratch healing rate, number of cell invasions, and expression of DUSP1 in the FA+OSM group were obviously decreased (P<0.05); the apoptosis rate, the methylation level of DUSP1, the expression of p38 MAPK protein, and the phosphorylation level of ERK were obviously increased (P<0.05). Compared with the FA+OSM group, the cell OD_450_ values (48, 72 h), scratch healing rate, number of cell invasions, and expression of DUSP1 in the FA+OSM+DAC group were obviously increased; the apoptosis rate, the methylation level of DUSP1, the expression of p38 MAPK protein, and the phosphorylation level of ERK were obviously reduced (P<0.05). **Conclusion** FA may inhibit DUSP1 expression by enhancing DUSP1 methylation, regulate downstream MAPK signal pathway, then promote apoptosis, inhibit cell invasion and metastasis, and ultimately reduce OSM resistance in NSCLC cells.

在我国，肺癌的发病率和死亡率正在不断上升，调查显示，2020年肺癌新发病例和死亡病例分别约为82和72万例，位居所有癌症的首位^[1]^。非小细胞肺癌（non-small cell lung cancer, NSCLC）是最常见的肺癌亚型，约占肺癌病例的85%以上，其恶性程度高，预后差，患者的5年生存率仅为15%^[2]^。临床对NSCLC的治疗主要有手术切除、化疗和放疗等方式，而对于晚期NSCLC患者主要采取化疗方式。奥西替尼（Osimertinib, OSM）是第三代表皮生长因子受体酪氨酸激酶抑制剂（epidermal growth factor receptor-tyrosine kinase inhibitors, EGFR-TKIs），其被应用于晚期NSCLC的靶向治疗，具有较好的治疗效果^[3,4]^。但若长期连续使用OSM，患者对OSM的敏感性将大幅降低，这限制了NSCLC的治疗效果^[5]^。OSM的耐药机制较为复杂，目前的研究还不深入，因此，探究OSM新的耐药机制，将有助于制定有针对性的治疗手段，从而有效解决耐药问题。双特异性磷酸酶1（dual specificity phosphatase 1, DUSP1）是MKP磷酸酶家族的重要成员，能广泛参与机体内信号传导、细胞生长发育，主要通过水解灭活丝裂原活化蛋白激酶（mitogen-activated protein kinase, MAPK）家族成员发挥负反馈调节作用^[6,7]^。研究^[8]^发现DUSP1参与了多种肿瘤的发生与发展，高表达的DUSP1可以促进NSCLC血管生成、侵袭和转移。然而，目前关于DUSP1甲基化与OSM耐药性关系的研究，还鲜有报道。叶酸（folic acid, FA）是一种可溶膳食性维生素，参与细胞的DNA甲基化、合成和修复，FA缺乏或代谢异常可导致DNA低甲基化，进而引起肿瘤或其他疾病的发生^[9,10]^。但FA在NSCLC组织或细胞中对DUSP1的调节作用和对OSM耐药性的影响未见报道。地西他滨（Decitabine, DAC）是一种DNA甲基转移酶抑制剂，其触发去甲基化导致体外和体内表观遗传沉默的肿瘤抑制基因的连续再激活。本研究建立OSM耐药细胞株PC9R，探究FA调控DUSP1甲基化对PC9R细胞OSM耐药性的影响。

## 1 资料与方法

### 1.1 细胞

NSCLC OSM敏感细胞株PC9购自中国科学院细胞库。

### 1.2 主要试剂

FA（货号：F8758）购自美国Sigma公司；OSM（货号：128830）购自瑞典AstraZeneca AB公司；DAC（国药准字H20140050）购自齐鲁制药公司；CCK-8试剂盒（货号：CK04）购自日本东仁化学科技有限公司；Annexin V-FITC/PI细胞凋亡双染检测试剂盒（货号：556507）购自美国BD生物科学有限公司；Transwell小室（货号：3450）购自Corning公司；BCA蛋白定量试剂盒（货号：PC0020）购自北京索莱宝科技有限公司；聚合酶链式反应（polymerase chain reaction, PCR）反转录试剂盒（货号：RR036A）购自日本Takara公司；Trizol试剂（货号：15596018）购自美国Invitrogen公司；EZ DNA Methylation-Gold^TM^ Kit（货号：D5005）购自ZYMO Research公司；DUSP1（货号：ab61201）、p38 MAPK（ab182453）、细胞外调节蛋白激酶（extracellular regulated protein kinase, ERK）（货号：ab184699）、p-ERK（货号：ab201015）、GAPDH（货号：ab9485）一抗及其二抗（货号：ab6721）购自美国Abcam公司。

### 1.3 PC9R细胞株的建立

采用浓度梯度递增法建立OSM耐药的NSCLC耐药细胞株PC9R^[11]^。

### 1.4 细胞分组与处理

将PC9R细胞分为对照组、OSM组（5 μmol/L OSM）、FA组（600 nmol/L FA）、甲基化抑制剂DAC组（10 μmol/L DAC）、FA+OSM组（600 nmol/L FA+5 μmol/L OSM）和FA+OSM+DAC组（600 nmol/L FA+5 μmol/L OSM+10 μmol/L DAC）。细胞分组后加入相应浓度药物进行处理。

### 1.5 CCK-8法测定细胞增殖

于96孔板中接种PC9R细胞（5×10^3^个/孔），培养24 h后按1.4所述加入相应浓度药物，每组设置6个复孔。置含5%CO_2_的细胞培养箱中继续培养24、48、72 h后，加入CCK-8溶液（10 μL/孔），培养2 h。用酶标仪于450 nm处检测各孔吸光度值。

### 1.6 划痕实验检测细胞迁移

于6孔板中接种PC9R细胞（2×10^5^个/孔），并按1.4方法进行处理。培养24 h后，用200 μL无菌移液枪枪头在各孔垂直画出一道划痕，之后用无菌PBS洗涤细胞。加入新鲜培养基继续培养48 h，分别于0和48 h时用倒置显微镜观察并拍照，用Image J软件分析计算细胞划痕愈合率。划痕愈合率=（1-48 h的面积/0 h的面积）×100%。

### 1.7 Transwell实验检测细胞侵袭

收集1.4中的各组细胞，于Transwell小室上室（基质胶包被）中接种细胞（3×10^5^个/孔）。将600 μL完全培养基（含10%胎牛血清）加入下室，孵育24 h后，将穿膜细胞用4%多聚甲醛固定20 min，结晶紫染色15 min。光学显微镜计数穿膜细胞。

### 1.8 流式细胞术检测细胞凋亡

将PC9R细胞以5×10^4^个/孔的密度接种于6孔板中，培养24 h后按1.4方法进行处理。培养24 h后，收集各组细胞，根据Annexin V-FITC/PI双染细胞凋亡检测试剂盒说明书，于离心管中加入1×10^6^个细胞，并加入Binding Buffer（500 μL）悬浮细胞，于室温下分别加入5 μL Annexin V-FITC和5 μL PI避光反应15 min。流式细胞仪测定细胞凋亡。

### 1.9 实时荧光定量PCR（real-time fluorescence quantitative PCR, RT-qPCR）法测定细胞DUSP1

mRNA表达 Trizol试剂提取各组PC9R细胞总RNA，反转录制备cDNA后进行PCR扩增。引物序列设计如下：DUSP1上游引物：5’-GAGCTGTGCAGCAAACAGTC-3’，下游引物：5’-GTCTGCCTTGTGGTTGTCCT-3’；β-actin上游引物：5’-CCCTGGCACCCAGCAC-3’，下游引物：5’- GCCGATCCACACGGAGTAC-3’。用2^-ΔΔCt^方法计算DUSP1 mRNA的相对表达，β-actin为内参。

### 1.10 甲基化特异性PCR（methylation specific PCR, MSP）检测细胞中DUSP1启动子区甲基化状态

提取各组细胞DNA，各取1 μg进行亚硫酸盐修饰。MSP的反应体系为20 μL，正向引物：5’-TGTTTGGTAGGGCGGGTGA-3’，反向引物：5’-GTCGCACACAACCCAAATA-3’。PCR产物（范围=chr5:172198165-172198336，171 bp）位于DUSP1启动子和外显子1边界的CpG岛IV上。反应条件为：95^o^C 5 min，95^ o^C 15 s，58^ o^C 15 s，72^ o^C 15 s，循环35次，72^ o^C 10 min。取各组扩增产物6 μL进行琼脂糖凝胶电泳，使用BioQ软件分析甲基化程度。

### 1.11 Western blot分析各组细胞DUSP1蛋白、MAPK信号通路关键蛋白的表达水平

取对数生长期的PC9R细胞按上述的1.4处理。收集细胞，加入RIPA裂解液提取细胞蛋白质。采用BCA蛋白定量试剂盒对总蛋白浓度进行定量。取20 μg蛋白上样，经10% SDS-PAGE凝胶电泳分离，转膜1.5 h，4 ^o^C摇床上与5%脱脂牛奶封闭2 h。4 ^o^C下加入DUSP1（1:1000）、p38 MAPK（1:1000）、p-ERK（1:1000）、ERK（1:1000）、GAPDH（1:1000）一抗孵育24 h。于室温下加入相应二抗（1:5000）孵育1 h，ECL化学发光法显色，以GAPDH蛋白作为内参。进行曝光并拍照，采用Image Lab软件对目标蛋白的灰度值进行定量分析。

### 1.12 统计分析

本研究用Graph Pad Prism 7.0软件进行统计分析。计量数据表示为均数±标准差，采用单因素方差分析和LSD-t检验用于多组间比较。P<0.05为差异有统计学意义。

## 2 结果

### 2.1 FA对细胞增殖能力的影响

如图1所示，与对照组相比，OSM组细胞OD_450_值（48、72 h）显著下降（P<0.05），FA组无统计学差异（P>0.05），DAC组细胞OD_450_值（48、72 h）显著上升（P<0.05）。与OSM组相比，FA+OSM组细胞OD_450_值（48、72 h）显著下降（P<0.05）。与FA+OSM组相比，FA+OSM+DAC组细胞OD_450_值（48、72 h）显著上升（P<0.05）。

**图1:**
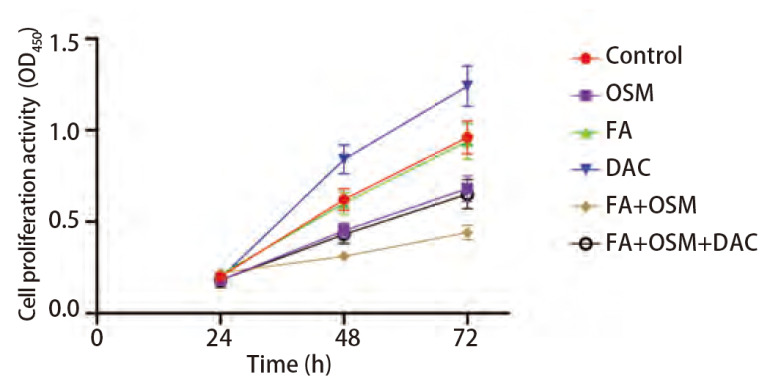
FA对PC9R细胞增殖能力的影响

### 2.2 FA对细胞迁移能力的影响

如图2所示，与对照组相比，OSM组细胞划痕愈合率显著下降（P<0.05），DAC组细胞划痕愈合率显著上升（P<0.05）。与OSM组相比，FA+OSM组细胞划痕愈合率显著下降（P<0.05）。与FA+OSM组相比，FA+OSM+DAC组细胞划痕愈合率显著上升（P<0.05）。

**图2:**
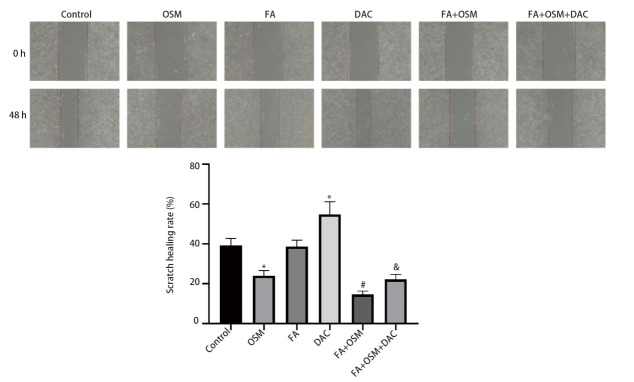
FA对PC9R细胞迁移能力的影响（划痕实验）。与对照组相比，*P<0.05；与OSM组相比，^#^P<0.05；与FA+OSM组相比，^&^P<0.05。

### 2.3 FA对细胞侵袭能力的影响

如图3所示，与对照组相比，OSM组细胞侵袭数目显著下降（P<0.05），FA组无统计学差异（P>0.05），DAC组细胞侵袭数目显著上升（P<0.05）。与OSM组相比，FA+OSM组细胞侵袭数目显著下降（P<0.05）。与FA+OSM组相比，FA+OSM+DAC组细胞侵袭数目显著上升（P<0.05）。

**图3:**
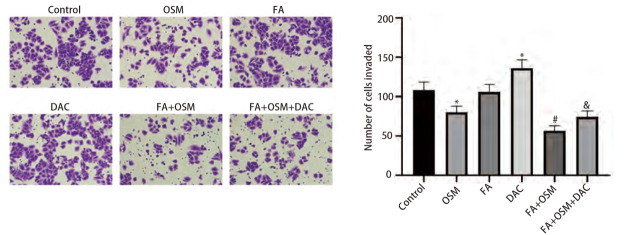
FA对PC9R细胞侵袭能力的影响（Transwell实验）。与对照组相比，*P<0.05；与OSM组相比，^#^P<0.05；与FA+OSM组相比，^&^P<0.05。

### 2.4 FA对细胞凋亡的影响

如图4所示，与对照组相比，OSM组细胞凋亡率显著上升（P<0.05），DAC组细胞凋亡率显著下降（P<0.05）。与OSM组相比，FA+OSM组细胞凋亡率显著上升（P<0.05）。与FA+OSM组相比，FA+OSM+DAC组细胞凋亡率显著下降（P<0.05）。

**图4:**
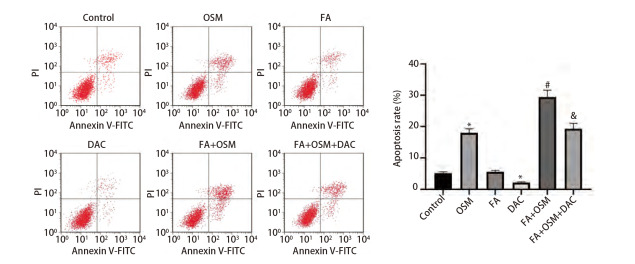
FA对PC9R细胞凋亡的影响。与对照组相比，*P<0.05；与OSM组相比，^#^P<0.05；与FA+OSM组相比，^&^P<0.05。

### 2.5 FA对细胞DUSP1 mRNA表达的影响

如图5所示，与对照组相比，OSM组细胞DUSP1 mRNA表达显著下降（P<0.05），FA组细胞DUSP1 mRNA表达无统计学差异（P>0.05），DAC组细胞DUSP1 mRNA表达显著上升（P<0.05）。与OSM组相比，FA+OSM组细胞DUSP1 mRNA表达显著下降（P<0.05）。与FA+OSM组相比，FA+OSM+DAC组细胞DUSP1 mRNA表达显著上升（P<0.05）。

**图5:**
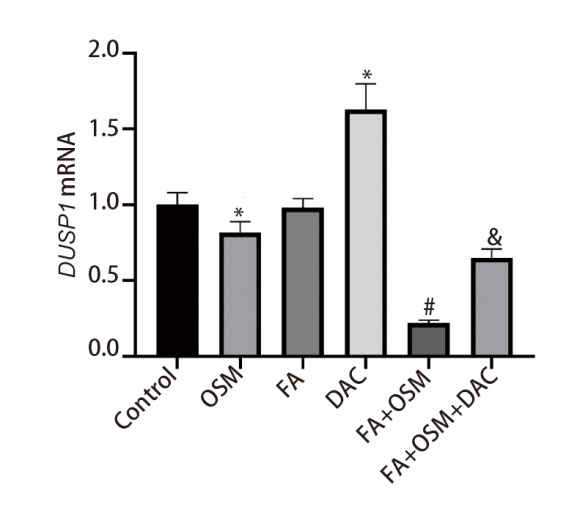
各组PC9R细胞DUSP1 mRNA表达比较。与对照组相比，*P<0.05；与OSM组相比，^#^P<0.05；与FA+OSM组相比，^&^P<0.05。

### 2.6 FA对DUSP1启动子区甲基化状态的影响

如图6所示，与对照组相比，OSM组细胞DUSP1甲基化水平显著上升（P<0.05），FA组无统计学差异（P>0.05），DAC组细胞DUSP1甲基化水平显著下降（P<0.05）。与OSM组相比，FA+OSM组细胞DUSP1甲基化水平显著升高（P<0.05）。与FA+OSM组相比，FA+OSM+DAC组细胞DUSP1甲基化水平显著下降（P<0.05）。

**图6:**
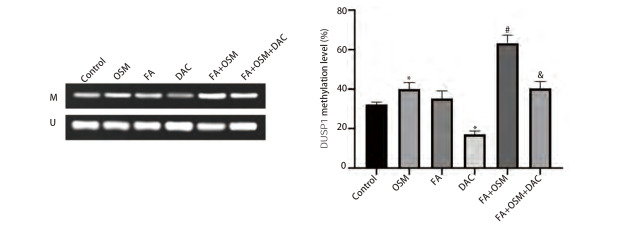
MSP检测PC9R细胞DUSP1甲基化状态。与对照组相比，*P<0.05；与OSM组相比，^#^P<0.05；与FA+OSM组相比，^&^P<0.05。

### 2.7 FA对细胞DUSP1蛋白、DUSP1下游MAPK信号通路关键蛋白表达水平的影响

如图7所示，与对照组相比，OSM组细胞DUSP1蛋白表达显著下降，p38 MAPK蛋白、ERK磷酸化水平显著上升（均P<0.05）；FA组无统计学差异（P>0.05）；DAC组细胞DUSP1蛋白表达显著上升，p38 MAPK蛋白、ERK磷酸化水平显著下降（均P<0.05）。与OSM组相比，FA+OSM组细胞DUSP1蛋白表达显著下降，p38 MAPK蛋白、ERK磷酸化水平显著上升（均P<0.05）。与FA+OSM组相比，FA+OSM+DAC组细胞DUSP1蛋白表达显著上升，p38 MAPK蛋白、ERK磷酸化水平显著下降（均P<0.05）。

**图7:**
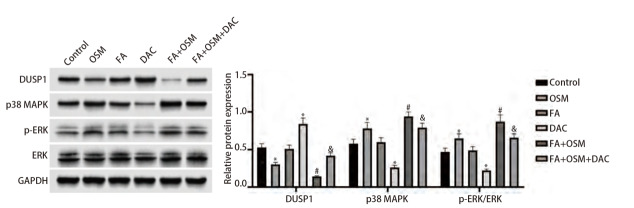
FA对细胞DUSP1蛋白、DUSP1下游MAPK信号通路关键蛋白表达水平的影响。与对照组相比，*P<0.05；与OSM组相比，^#^P<0.05；与FA+OSM组相比，^&^P<0.05。

## 3 讨论

NSCLC是一种发病率和死亡率逐年上升的恶性肿瘤疾病，发病前期确诊较困难，一般发现时患者已处于中晚期，给治疗带来较大的阻碍^[12,13]^。NSCLC的发病原因主要有环境因素、吸烟、职业暴露、辐射、既往肺部感染、遗传等，其中吸烟是引起NSCLC的最主要原因^[14,15]^。OSM是治疗NSCLC的一线药物，是一种靶向治疗药物，约50%的NSCLC患者存在EGFR突变，OSM可以有效延长患者的生存时间，但存在OSM耐药性问题，长期使用OSM会使OSM的疗效明显下降^[16,17]^。因此，本研究通过建立OSM耐药细胞株PC9R，探究FA对PC9R细胞OSM耐药性的影响。

DUSP1是本研究组前期通过建立两种NSCLC OSM耐药细胞株（PC9R、H1975R），并通过高通量基因芯片分析了差异化基因表达谱所发现的基因。研究^[18⇓-20]^发现，DUSP1可以参与调控多种肿瘤的发生与发展，其在晚期乳腺癌、NSCLC、胰腺癌中的表达水平显著升高。Chen等^[21]^研究发现DUSP1的异位表达可以通过影响ERK信号通路而增加化疗药物吉非替尼敏感性。Li等^[22]^研究发现DUSP1在三阴性乳腺癌中的表达受其启动子甲基化调节。本研究结果显示，敏感株中抑制DUSP1甲基化可增强其表达，并减弱OSM对敏感株的杀伤力。

FA是一种水溶性B族维生素，在体内以四氢叶酸的形式存在。四氢叶酸可以用于嘌呤、胸苷酸的合成以及蛋氨酸循环途径中同型半胱氨酸的甲基化^[23]^。在肿瘤发生的早期，DNA甲基化水平降低，局部CpG岛甲基化程度升高，广泛调控原癌基因和抑癌基因的表达，是肿瘤发生发展的重要机制之一^[24]^。张银铃等^[25]^研究发现一定浓度的FA可以提高卵巢癌细胞OV90基因组的甲基化水平，对其生长具有一定抑制作用。通过检索DrugBank数据库，发现FA在小鼠肌肉组织中可增强DUSP1启动子区甲基化而减弱其表达^[26]^。本研究结果显示，与OSM组相比，FA+OSM组细胞OD_450_值（48、72 h）、划痕愈合率、细胞侵袭数目、DUSP1水平显著下降，细胞凋亡率、DUSP1甲基化水平、p38 MAPK蛋白表达、ERK磷酸化水平显著升高。与FA+OSM组相比，FA+OSM+DAC组细胞OD_450_值（48、72 h）、划痕愈合率、细胞侵袭数目、DUSP1水平显著升高，细胞凋亡率、DUSP1甲基化水平、p38 MAPK蛋白表达、ERK磷酸化水平显著降低。这说明FA与OSM联用可能通过增强DUSP1甲基化抑制DUSP1表达，调控下游MAPK信号通路，抑制NSCLC细胞OSM耐药性。

综上所述，FA与OSM联用可能通过增强DUSP1甲基化抑制DUSP1表达，抑制NSCLC细胞OSM耐药性。本研究为临床治疗中改善NSCLC患者化疗耐药性提供了科学依据和参考。但本研究是在细胞水平上进行的分析，还需进一步进行相关动物试验及临床试验的深入探索。

**Competing interests**

The authors declare that they have no competing interests.

**Author contributions**

Liu L conceived the project and supervised the experiments. He WJ conducted the experiments and performed the data analysis. Both the authors read and approved the final manuscript.
